# How Contemporary Disney Film Can Be Used for Mental Health Teaching in Schools: A Case Study of *Inside Out (2015)*

**DOI:** 10.1192/bjo.2022.115

**Published:** 2022-06-20

**Authors:** Charlotte Caves, Robin Basu-Roy

**Affiliations:** Imperial College London, London, United Kingdom

## Abstract

**Aims:**

Mental health disorders can be a burden on both patients and the National Health Service. With the majority of lifetime mental health problems emerging in childhood and the prevalence of childhood mental illness increasing, the need for effective, standardised mental health education and fostering healthy socio-emotional development is more important than ever before. The aim was to explore if *Inside Out* provides an accurate representation of depression, and thus, can it be a useful resource for teaching mental health and developing emotional awareness in the classroom?

**Methods:**

I explored a novel educational concept: ‘edutainment’, to see if it has use in state mental health education. This project provides a quantitative coding analysis and a qualitative artistic analysis of a contemporary Disney film, Inside Out (2015), for The International Classification of Diseases 10th Edition (ICD-10) depression symptoms. Depression has been chosen as an example of a mental health disorder as it is one of the commonest mental health problems and the leading cause of disability worldwide.

**Results:**

Inside Out provides an accurate representation of many of the ICD-10 ‘core’ and ‘cognitive’ symptoms of depression through both coding words and artistic means.

**Conclusion:**

*Inside Out*, alongside teacher-led discussion, could be useful in teaching children about depression in a relaxed but educational way. *Inside Out* features themes that can help children develop their emotional intelligence and reduce mental health stigma. I highlight a need for standardised mental health education and suggest that film may be an effective tool for learning about common mental health conditions, such as depression.

